# Supplementary material for: Platicon microcomb generation using laser self-injection locking
Source: Nat Commun. 2022 Apr 1;13:1771. doi: 10.1038/s41467-022-29431-0 (PMC8975808; doi:10.1038/s41467-022-29431-0)
Supplement: Supplementary file 1 — Supplementary Information [file 41467_2022_29431_MOESM1_ESM.pdf]

Supplementary Information for:
Platicon microcomb generation using laser self-injection locking

Grigory Lihachev,[1,*] Wenle Weng,[1,2,*] Junqiu Liu,[1,*] Lin Chang,[3] Joel Guo,[3] Jijun He,[1]
Rui Ning Wang,[1] Miles H. Anderson,[1] Yang Liu,[1] John E. Bowers,[3] and Tobias J. Kippenberg[1]

[1] *Institute of Physics, Swiss Federal Institute of Technology Lausanne (EPFL), CH-1015 Lausanne, Switzerland*
[2] *Present address: Institute for Photonics and Advanced Sensing (IPAS), and School of Physical Sciences, The University of Adelaide, Adelaide, South Australia 5005, Australia*
[3] *ECE Department, University of California Santa Barbara, Santa Barbara, CA 93106, USA*

SUPPLEMENTARY NOTE 1: NUMERICAL SIMULATION OF A SEMICONDUCTOR-LASER-MICRORESONATOR SYSTEM BASED ON COUPLED EQUATIONS

To analyze the dynamics of the laser-microcomb system, we develop a model where an injection-locked semiconductor laser is coupled to counter-propagating fields in a Kerr microresonator. The model comprises four coupled equations that are written as:

$$\frac{dN}{dt} = \frac{I}{e} - \gamma N - a(N - N_0)|A_\mathrm{L}|^2 \tag{1}$$

$$\frac{dA_\mathrm{L}}{dt} = \left[\frac{(1-i\alpha)}{2}(a(N-N_0) - \frac{1}{\tau}) - i\omega_{\mathrm{L0}}\right] A_\mathrm{L} + \kappa_\mathrm{inj} e^{i\theta} A_\mathrm{ccw} \tag{2}$$

$$\frac{\partial A_\mathrm{cw}}{\partial t} = i\frac{D_2}{2}\frac{\partial^2 A_\mathrm{cw}}{\partial \phi^2} + ig(|A_\mathrm{cw}|^2 + 2|A_\mathrm{ccw}|^2)A_\mathrm{cw} - \frac{\kappa}{2}A_\mathrm{cw} + i\delta A_\mathrm{ccw} + \kappa_\mathrm{inj} e^{i\theta} A_\mathrm{L} \tag{3}$$

$$\frac{dA_\mathrm{ccw}}{dt} = ig\left(|A_\mathrm{ccw}|^2 + 2\int_0^{2\pi} \frac{|A_\mathrm{cw}|^2}{2\pi}\, d\phi\right) A_\mathrm{ccw} - \frac{\kappa}{2}A_\mathrm{ccw} + i\delta^* A_{\mathrm{cw},\mu=0} \tag{4}$$

Equations 1 and 2 are the semiconductor laser rate equations [1–3], where A_L represents the complex field of the laser and $|A_\mathrm{L}|^2$ is the photon number in the laser cavity, I is the bias current, N is the carrier number in the laser cavity, α is the Henry factor, a is the differential gain, N_0 is the carrier number at transparency, γ is the carrier recombination rate, τ is the laser photon lifetime, ω_{L0} is the resonant frequency of the laser cavity mode relative to the pumped microresonator mode frequency, and e is the elementary charge.

Using A_L as the driving field, Equation 3 is a modified Lugiato-Lefever equation (LLE) [4] for the computation of the slowly varying clockwise (cw) field A_cw, while Equation 4 describes the dynamics of the counterclockwise (ccw) field A_ccw. Moreover, $|A_\mathrm{cw}|^2$ and $|A_\mathrm{ccw}|^2$ are the intracavity photon numbers for the Kerr microresonator system. In these equations, ϕ is the azimuthal angle along the circumference of the rotating frame, D_2 is the second-order dispersion coefficient, κ is the microresonator loaded photon decay rate, g is the single-photon-induced frequency shift due to the Kerr effect, and δ is the backscattering-induced linear coupling rate between the cw and the ccw modes.

Since the power in the ccw mode is usually below the parametric four-wave-mixing threshold, here we treat the ccw field as ϕ-independent, and only the field in the central mode of the microcomb ($A_{\mathrm{cw},\mu=0}$) is scattered back to A_ccw. As a result, while the cross-phase modulation effect caused by the cw modes to the ccw modes takes the full power of the cw field profile into account with the integration $\int_0^{2\pi} \frac{|A_\mathrm{cw}|^2}{2\pi} d\phi$, the cross phase modulation caused by the ccw modes is simplified by using $|A_\mathrm{ccw}|^2$. The ccw field is injected back into the laser, with a feedback phase shift θ and a coupling rate between the microresonator mode and the laser mode that is denoted by $\kappa_\mathrm{inj} = \sqrt{\kappa_\mathrm{ex}\kappa_\mathrm{Lo}}$, where κ_ex is the external coupling rate of the microresonator, and κ_Lo is the out-couple rate of the laser.

The numerical simulation is carried out with the Runge-Kutta method, with the values of parameters set as $I = 250\,\mathrm{mA}$, $V = 20 \times 10^{-17}\,\mathrm{m}^3$, $\gamma = 1 \times 10^9\,\mathrm{Hz}$, $a = 1 \times 10^4\,\mathrm{Hz}$, $N_0 = Vq_0 = 2 \times 10^8$ (where $V = 20 \times 10^{-17}\,\mathrm{m}^3$ is the laser cavity volume and $q_0 = 1 \times 10^{24}\,\mathrm{m}^{-3}$ is the carrier density at transparency), $\alpha = 5$, $\tau = 5 \times 10^{-12}\,\mathrm{s}$, $\kappa/2\pi = 100\,\mathrm{MHz}$, $\kappa_\mathrm{ex}/2\pi = 50\,\mathrm{MHz}$, $\delta/2\pi = 10\,\mathrm{MHz}$, $\theta = \pi/2$, $\kappa_\mathrm{Lo}/2\pi = 4.6\,\mathrm{GHz}$, $g = 0.56\,\mathrm{rad/s}$, $D_1/2\pi = 26\,\mathrm{GHz}$,

and $D_2/2\pi = -60$ kHz. Complex random noise is added into both the laser field and the microresonator intracavity fields to initiate the lasing and the platicon formation. Numerical integration of the coupled equations yields the results presented in Figure 5 in the main article. The sweeping time is $3.6\,\mu$s in lab time, corresponding to 360 microresonator photon decay times. Such a long sweeping time eliminates transient instabilities after the platicon is created, allowing us to confirm the high stability and the coherence of the platicon structure.

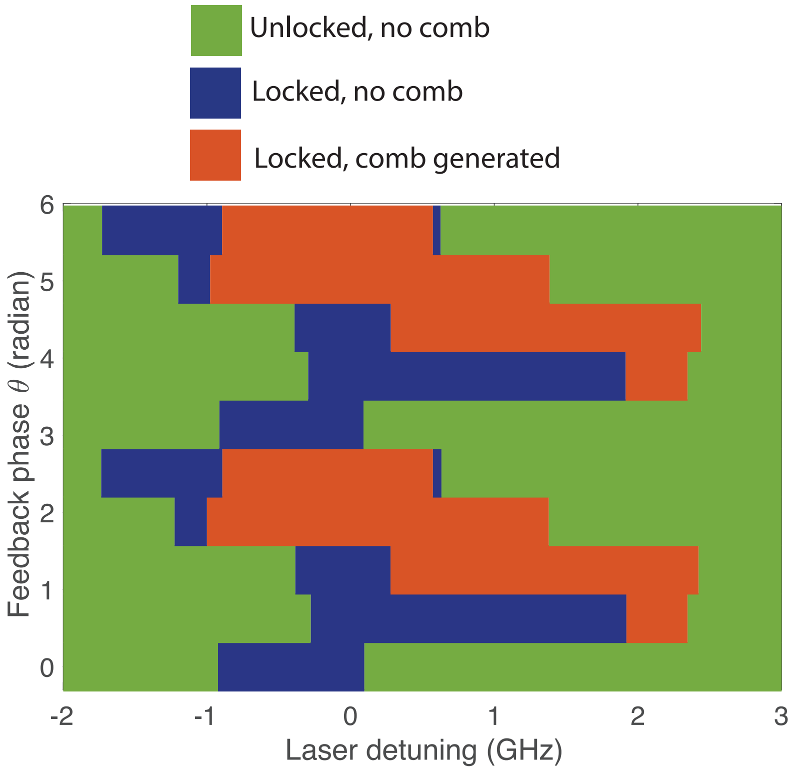

Supplementary Figure 1. **Impact of feedback phase on the laser-microresonator system state.** Laser cavity resonance down-sweeping across a microresonator resonance is repeated with the feedback phase θ changing from 0 to 2π. Based on the laser frequency and the microresonator intracavity field profile, the simulation results are categorized into three different groups denoted by three different colors.

We vary the value of θ in the model from 0 to 2π with a step size of 0.2π and repeat the simulation of the laser detuning sweeping using the same procedure as the simulation in the main text. Supplementary Figure 1 shows the impact of the optical feedback phase on the state of the laser-microresonator system. In the 2-D parameter space, we categorize the results into three states, including: (1, in green) the laser is unlocked and no microcomb is generated, (2, in blue) the laser is self-injection locked without producing microcomb, and (3, in red) the laser is self-injection locked and stable platicon microcomb is generated. Although this simulation is highly qualitative, one can see the critical importance of choosing a proper feedback phase as with some phase values no comb can be generated. We also note that, with certain phase values, while the microcomb existence range is very narrow, the laser can be self-injection locked for a relatively broad laser-microresonator detuning range. One may find it useful to develop compact high-power self-injection locked laser systems without exciting optical nonlinear generation. Moreover, the simulation results display a θ-dependent pattern with a period of π, which is caused by the back-and-forth travelling of the laser field that enables the self-injection locking.

SUPPLEMENTARY NOTE 2: SIMULATION OF THE FORMATION OF PERFECT PLATICON CRYSTAL STATES

A surprising experimental observation is that perfect platicon crystal states (i. e., multi-platicon states with equal inter-platicon separations) can often be generated. We postulate that, just like the formation of perfect DKS crystals, the local dispersion deviation caused by mechanisms such as mode-interaction-induced avoided mode crossing can introduce intracavity periodic optical lattices during the inception stage of the platicons, thus leading to the formation

of equally spaced multi-platicon states. To test our postulation, when setting up the microresonator resonance dispersion, we add an extra resonance frequency shift of around 5 MHz to a single resonance. The magnitude of this frequency shift is in qualitative agreement with the resonance frequency deviations shown in Figure 1 (d) in the main article. The platicon formation simulation is repeated with the mode number of the frequency-shifted mode changing from -2 to -5. Supplementary Figure 2 shows the configured dispersion with the frequency-shifted mode of $\mu = -2$. With the change of the frequency shifted mode number, the intracavity optical lattice period number is varied from 2 to 5. Supplementary Figure 3 summarizes the simulation results. From top to bottom, each panel corresponds to a lattice period number, arranged in ascending order. We see that perfect platicon crystals with composite platicon numbers equal to the lattice period numbers are indeed generated, and the microcomb spectra also show the multi-FSR line spacing, which are in qualitative agreement with our experimental observations. Since local resonance deviations of some amount always exist in practice, and they can be tuned with temperature change of the microresonator, perfect platicon crystals with distinct composite platicon numbers can be generated in the same microresonator which is exactly what we observe in experiments. Also, the simulated platicons exhibit a relative motion along the ϕ direction. Such a platicon group velocity shift is caused by the recoil effect due to the intensity increase in the frequency-shifted resonances.

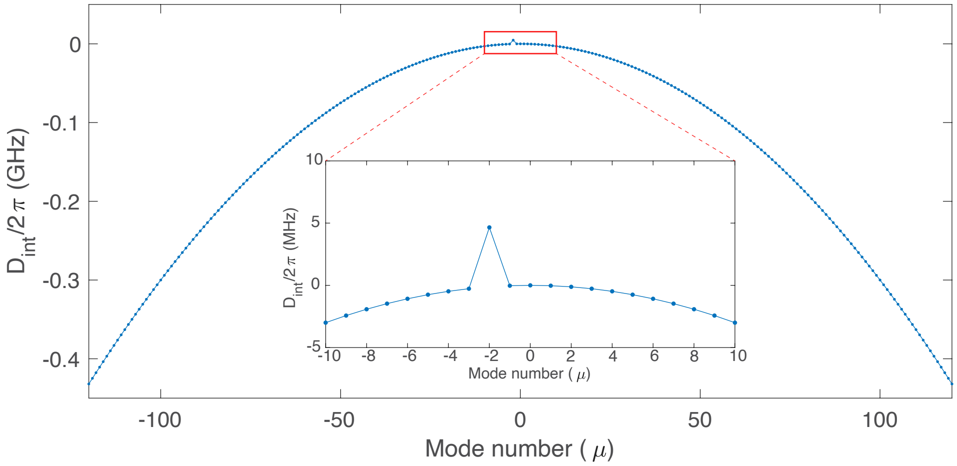

Supplementary Figure 2. Resonance frequency deviation for perfect platicon crystal formation. The mode resonance of $\mu = -2$ is frequency shifted by 5 MHz to introduce the local resonance frequency deviation from the D_2-only dispersion curve.

SUPPLEMENTARY NOTE 3: EXPERIMENTAL OBSERVATION OF IMPACT OF COUPLING GAP VARIATION

Supplementary Figure 4 presents the laser bias current sweeping spectroscopy results with slightly varied gap between the laser and the photonic chip. Since we are not able to determine the gap in the experiment, we cannot know the feedback phase of the backscattered field that enables the self-injection locking. The purpose of this figure is to show that the feedback phase change not only significantly influences the range of the self-injection locked state, but also has critical impact to whether the microcomb can be generated or not. These results corroborate the simulation results with varied feedback phase presented in the Supplementary Note 1.

SUPPLEMENTARY NOTE 4: CHARACTERIZATION OF THE PHOTONIC CHIP

In Supplementary Figure 5 we provide broadband measurements of the transmission and reflection spectra and statistics of the backreflection, intrinsic cavity loss, bus waveguide coupling rate and mode splitting rate obtained by fitting the transmission trace. Transmission trace calibration includes input and output lensed fiber losses. From the reflection trace, no clear dependence of backreflection rate is visible. The histogram shows that half of the resonances have a backreflection of 0.05 in power – the level of chip facets reflection. Still, more than 50 resonances exhibit backreflection coefficients of 0.1-0.15.

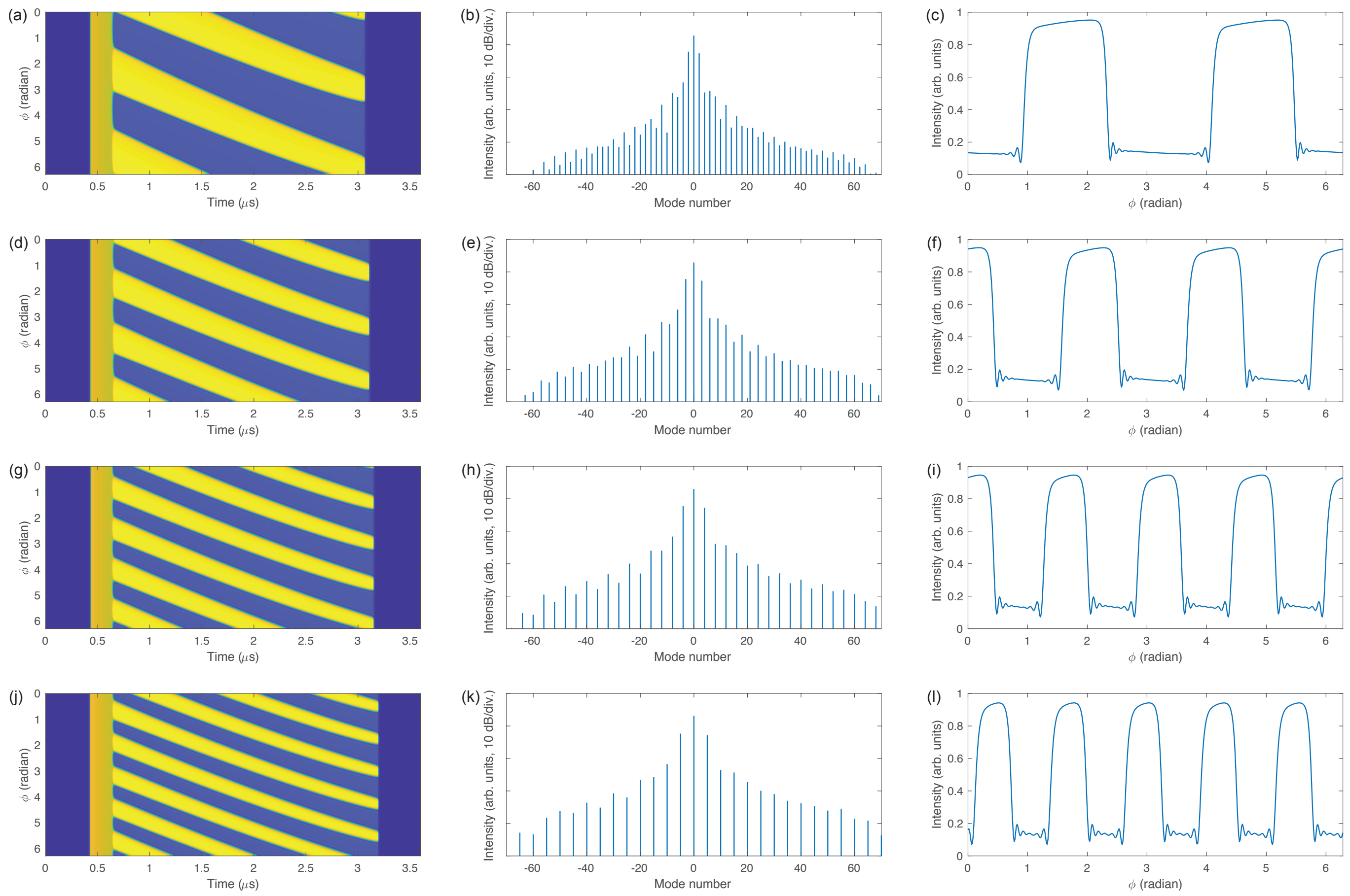

Supplementary Figure 3. **Simulation of perfect platicon crystals.** (a, d, g, j) Dynamical evolutions of the microresonator intracavity intensity as the laser cavity resonance frequency is swept downwards across a microresonator resonance. The imposed intracavity lattice period number is varied from 2 to 5, respectively. (b, e, h, k) The corresponding platicon comb spectra at the time of 2.1 μs. (c, f, i, l) The temporal profiles of the perfect platicon crystal states corresponding to the microcomb spectra in (b, e, h, k).

SUPPLEMENTARY REFERENCES

* These authors contributed equally to this work.

[1] Tarucha, S. & Otsuka, K. Response of semiconductor laser to deep sinusoidal injection current modulation. *IEEE Journal of Quantum Electronics* **17**, 810–816 (1981). URL https://ieeexplore.ieee.org/abstract/document/1071186.

[2] Kondratiev, N. *et al.* Self-injection locking of a laser diode to a high-q wgm microresonator. *Optics Express* **25**, 28167–28178 (2017).

[3] Weng, W. *et al.* Gain-switched semiconductor laser driven soliton microcombs. *Nature Communications* **12**, 1–9 (2021).

[4] Lugiato, L. A. & Lefever, R. Spatial dissipative structures in passive optical systems. *Physical review letters* **58**, 2209 (1987). URL https://journals.aps.org/prl/abstract/10.1103/PhysRevLett.58.2209.

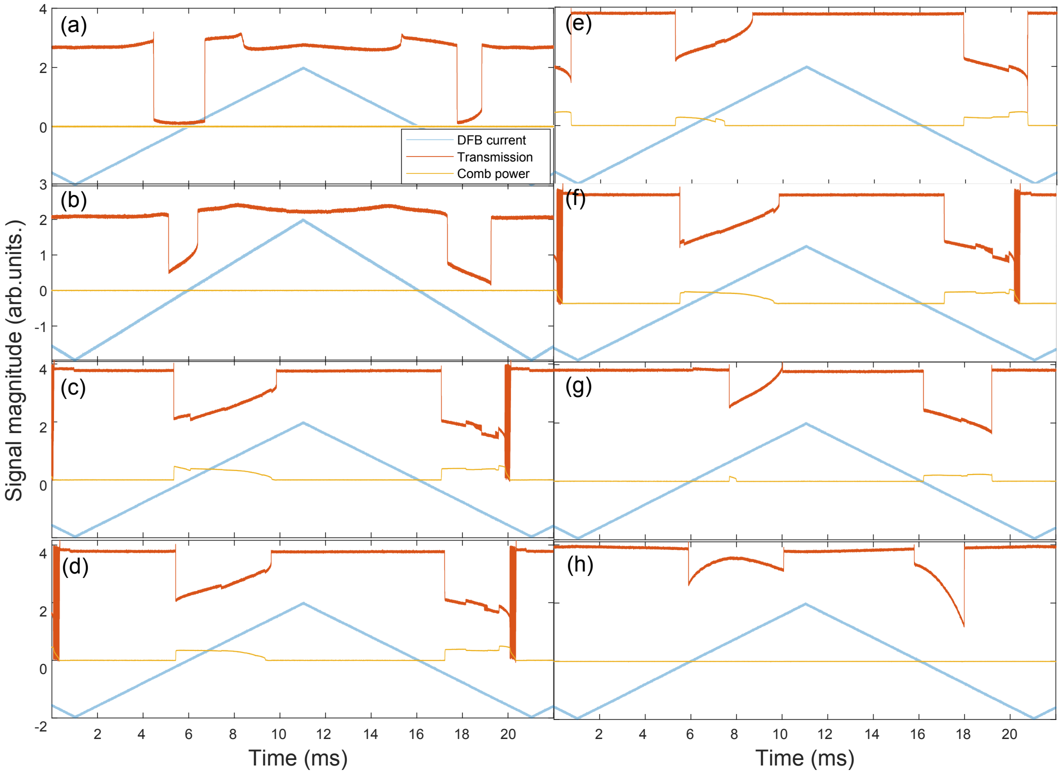

Supplementary Figure 4. **Impact of varied coupling gap as the laser bias current is swept.** Based on the transmission spectroscopy, the self-injection locking range can differ by nearly a factor of 4. Additionally, microcomb can only be generated in (c, d, e, f, and g).

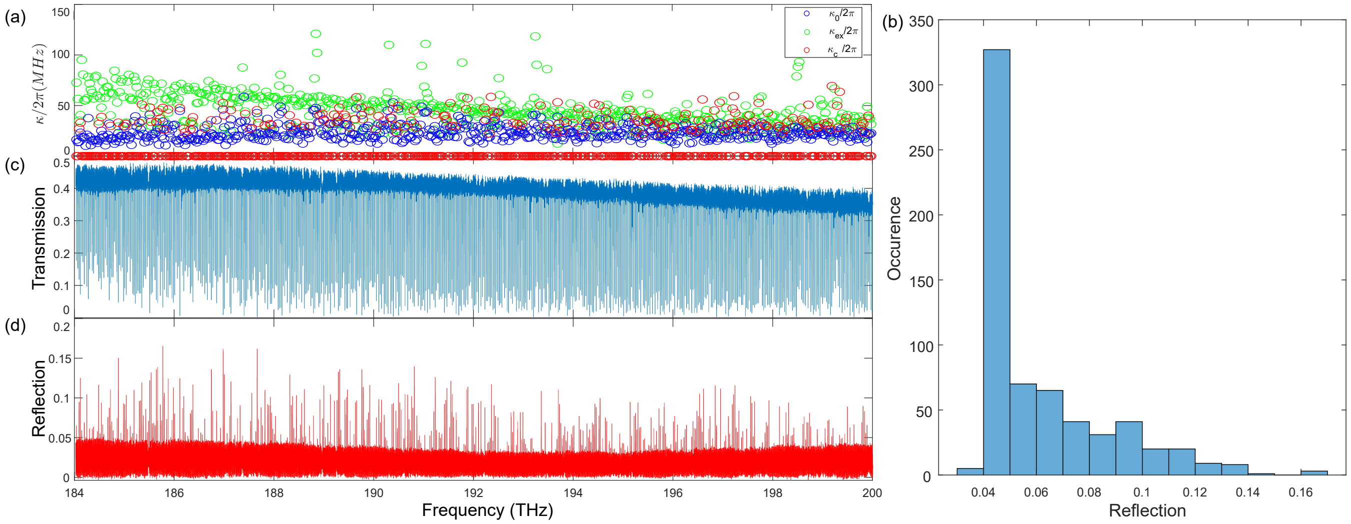

Supplementary Figure 5. **Linear spectroscopy for characterization of the Si_3N_4 photonic chip.** (a) Frequency-dependent microresonator intrinsic loss κ_0, bus waveguide coupling κ_{ex} and mode splitting rate κ_c. b) Histogram of backreflection values of the cavity resonances. (c) Transmission and (d) reflection traces of the microresonator with an FSR of 26.2 GHz.
